# Systemic sexism in academia: an early-career viewpoint

**DOI:** 10.1093/biosci/biaf076

**Published:** 2025-06-16

**Authors:** Olivia del Giorgio, Gabriela Fontanarrosa, Silvia Lomáscolo, María Piquer-Rodríguez

**Affiliations:** Department of Geography at McGill University, in Montreal, Quebec, Canada; Institute of Neotropical Biodiversity at Tucumán National University and CONICET, in Tucumán, Argentina; Institute of Regional Ecology at Tucumán National University and CONICET, in Tucumán, Argentina; Institute of Geographical Sciences at Freie Universität, in Berlin, Germany

Over the past years, a theme has recurrently popped up in conversation among us (the present authors): Discussions over Zoom, at conferences, on bumpy roads in the field, during walks with the kids, over coffee, etc., have tended to steer onto the hardships of navigating systemic sexism in the early stages of our research careers. The space we have created for these discussions among ourselves has helped to ground our individual experiences in a collective reality. It is a testament to the importance of the lifeboat formed by connections between female academics and friends (Bentley and Garrett 2023). Yet a lifeboat is only that: It keeps us afloat but often barely.

The consensus we've reached is that having to build, inflate, and constantly patch up our own lifeboat as we navigate a slew of systemically produced, sexist challenges adds injustice to injustice. Ending misogyny in academia requires, first and foremost in our opinion, that we openly denounce the setbacks that we face, by virtue of our womanhood, starting at the very headwaters of our careers. So, in what follows, we share the main hurdles we and other women around us have faced, across institutions and continents, in the throes of our growth as researchers.

## Occupying academic space: A case of fostered intellectual insecurity

Academia poses its challenges for both men and women when it comes to overcoming imposter syndrome. Almost ironically, doing so is essential to a researcher thriving in the early stages of their career: taking on challenges, applying for funding, collaborating, and pursuing merit recognition opportunities all require intellectual confidence. Nurturing the self-assurance of early-career researchers is therefore a key element in their success and, by virtue, also that of the academic community. But our experiences point to the opposite often happening for women—a fostering of intellectual *insecurity* beginning early on in our academic trajectories. We cannot count the number of times male academics have assumed a position of intellectual superiority in conversations—“mansplaining”, for example, the complexities of a certain topic, why we have encountered issues in our methodologies, or how we should navigate risks in the field. Even if the offering is well informed or well intended, the tone matters: We are often spoken to in a way that assumes our ignorance, leading many of us to internalize a feeling of a lack of intellectual credibility. Along with being spoken *to* condescendingly, we are also spoken *of* differently from how our male peers are. For example, recognitions of our achievements recurrently focus on our success in performing socializing or organizational chores, negating or underestimating professional accomplishments related to funding, networking, and publication success (among others). And at the extreme but unfortunately not uncommon end of the relational spectrum are interactions involving gaslighting, thought and tone policing, sexual harassment, and extortion (see, e.g., Brown 2019, Fernando and Prasad 2019, and Täuber et al. 2022). From our experience, the greatest damage to our intellectual confidence has come from toxicity exhibited by people we admire or who hold power over our careers.

What these behaviors lead to is *impostorization* (and not just imposter syndrome; Gutierrez and Cole 2023) and to women researchers valuing themselves lower than male scientists in self-valuations, even when academic productivity is held constant (Lomáscolo et al. 2024). In brief, machismo—the norms and behaviors that promote male dominance, control, and the belief in the inherent superiority of men over women (Maffia 2007)—stands center stage in this tragedy of intellectual confidence.

## Door closed to the boys’ club

Machismo culture in academia also often results in women sitting out spaces of encouragement and collegiality because they are not allowed into the boys’ club. Where we have male colleagues and supervisors, and in particular where we are part of male-dominant teams, we often experience exclusion from a dynamic of male chumminess. This homophily among men, oftentimes unintended, affects both how we perceive belonging within academia and, concretely, how we are supported (or not supported) during the early stages of our careers relative to our male counterparts—for example, not being included in informal brainstorming, being less likely to be invited to speak at seminar series (Chuliver Pereyra et al. 2021), and male academics preferentially publishing with male over female colleagues (Grosso et al. 2021). This exclusion from male-dominated spaces of collegiality leads many of us to work double-time to make a space (and name) for ourselves (Fontanarrosa et al. 2024).

Along with taking on more responsibilities to “prove” ourselves to others, gaining academic respect can also involve earning extraordinary entry into the boys’ club—an acceptance often contingent on becoming the more publicly admired “badass” woman. For many of us, this implies emotional masking and therefore an added and constant background effort. Although the pressures of emotional masking are dissipating along with increased endorsement of empathic leadership styles, women have and still do suffer from it. Some then drag these learned and performative behaviors into the later stages of their careers, themselves becoming toxic mentors and colleagues (Rogers 2017). What we've seen is that the toxic behaviors of these senior female researchers are commonly targeted against the younger women (and not men) that they supervise and work with, ironically reproducing the patterns that lead to early-career dropout among women.

## Extra trials of fieldwork: Defending expenses and extra safety plans

For many researchers, the early stages of their careers in academia are marked by a heavy fieldwork component, which usually brings with it a set of logistical and personal challenges. Dealing with many long days far from home, equipment failures, and obstacles to data collection is the norm, forming part of the set of (often underacknowledged) competencies we develop as field-researchers. Adding to these, a simple fact stands: fieldwork is inherently riskier for women (and minority groups). On top of the baseline challenges of fieldwork, we, as women, face a slew of added challenges in the field that necessarily affect how we navigate data collection in the early stages of our careers and, most importantly, in what shape we end up in physically and emotionally after having done so. For one, when the risks of sexual harassment (and of rape and even murder; Carlin et al. 2023) are elevated, we have to address a series of logistical constraints: Should we be accompanied and, if so, by whom and under what circumstances? Are more frequent check-ins and tracking protocols necessary? Are our sleeping arrangements sufficiently secure (e.g., is it best to share quarters with an assistant or colleague? Does the room lock)? And when and where can we safely engage with different groups of people?

Adding to the logistical and emotional burden of these risk-minimizing considerations, we usually have to convince male colleagues and supervisors of their necessity. A pressure on hyper performance and productivity sometimes then sets in, as we feel the need to prove that the extra funds and time spent were, despite nothing terrible happening to our safety, worth it. Simultaneously, in the field, we still deal with the harms of sexist behavior that no amount of risk remediation (aside from not doing fieldwork…) can avoid: inappropriate innuendos (e.g., comments on our desirability), the loss of authority and credibility in the presence of male company, trailing gazes, being interrupted in conversation, and catcalling, among many others. Together, this all mounts to make fieldwork, a key component to the early stages of some researcher's trajectory, particularly taxing for us as women.

## Norms and roles: Expectations and emotional contradictions

Another set of hurdles dealt to women researchers in the early stages of their careers involves gendered expectations and the resulting emotional contradictions that stem from established patriarchal norms. Albeit progress made in the last decades, academic women continue to take on more domestic and care responsibilities than academic men (Morgan et al. 2021, Sümer and Eslen-Ziya 2023). The added energy spent thinking about and addressing day-to-day tasks, such as home maintenance and the care of dependents—dubbed *charge mental* (or “mental load”) by French feminist activists (Dean et al. 2022)—very much affects (still, yes, in 2025) the academic productivity of women, especially those in their early career stages (Shalaby et al. 2021). This gives way to an important internal contradiction: Many of us, at some point or other, perceive that our personal lives affect our careers and that, simultaneously, our careers interfere with our personal lives (Lomáscolo et al. 2024). And all the more so when the plan is to have children—a decision frequently taken during the early stages of our careers. In a nutshell, what these gendered expectations and care inequalities lead to are substantial efforts on women's part to keep up with both our personal and professional goals and, for some of us, a feeling that we have to make a choice between doing one well or doing the other well—but not both.

## Stop the leak!

The described hurdles, among others, mount to limit what should be a moment of discovery and creativity into a narrow trench of actions and reactions, that goes constricting as we progress as women through the early stages of our careers in academia. Those who come out on the other side of that early career squeeze then have to confront the challenges of ascension into senior positions—the tightrope act (Bentley and Garrett 2023)—while carrying with them the disadvantages afforded by those initial trials. The underrepresentation of women in senior positions then itself becomes a barrier to the career ascension of other women—a self-sustaining monster of sorts (Allen et al. 2021).

So, what we see as the solution: Ending misogyny in academia requires, perhaps first and foremost, that the hurdles faced by female early-career researchers be recognized, tackled, and eliminated. To do so, we see three areas where sustained action is necessary.

First, women's representation and leadership opportunities must be proactively strengthened. Doing so requires that the structures determining career advancement—from hiring to publishing—be made equitable, accessible, and transparent. Along with setting clear diversity targets in hiring and promotion (e.g., gender-balanced leadership quotas), institutions must ensure that selection committees are trained to recognize and counteract implicit biases, which continue to shape recruitment and promotion decisions even in institutions that formally endorse equity policies (Llorens et al. 2021). The same attention to bias must extend to funding and publishing by standardizing double blind reviews for grant applications and publications, ensuring that research is evaluated on the basis of quality and not researcher identity. However, equitable career advancement requires more than reforming evaluation processes; it also means recognizing the full scope of contributions that sustain academic institutions (Wijesingha and Ramos 2017). The invisible labor that disproportionately falls on women—mentorship, service work, and caregiving (Hands et al. 2024)—must be taken into consideration in hiring, evaluation, and promotion decisions. And as a last point of many more that could be made, we need leadership training and recognition programs to be formalized, so that early- and mid-career female researchers are provided with the mentorship and institutional backing necessary to advance into leadership positions.

Second, institutions must ensure that academic careers are structured in ways that do not systemically disadvantage women. Paid parental leave needs to be guaranteed for everyone, with equal leave time given regardless of gender, to prevent caregiving responsibilities from disproportionately falling on women. For these leave provisions to be effective, they must be accompanied by practical accommodations, including on-campus childcare, flexible schedules for caregivers, and funding support for fieldwork and conference travel. Likewise, adjusted teaching loads, remote work options, and modified research timelines must become routine institutional practices, rather than being treated as special accommodations.

Third, institutions must commit to fostering an inclusive and accountable academic culture. Antiharassment policies cannot remain empty statements; they must be clearly defined, strictly enforced, and supported by real accountability mechanisms. At the same time, diversity, equity, and inclusion (DEI) programs must be integrated into institutional priorities—not as superficial branding exercises but as long-term commitments. These initiatives should include active bystander training and cultural competency education to prevent aggressive communication and discrimination from being normalized in academic spaces. All that said, true change requires more than policy and programming; it demands a shift in academic culture towards genuine allyship and everyday accountability. The barriers women continue to face must not only be acknowledged in institutional frameworks but actively challenged in our daily interactions. This means fostering an environment where women are supported and empowered in both formal and informal settings—whether in meetings, conferences, or discussions over coffee. True allyship goes beyond symbolic gestures; it means ensuring women are heard, credited for their work, and backed when they challenge the status quo. It means inviting, rather than expecting, us to take up space.

Together, all of this amounts to a process of accommodation that comes not from victimization and pitying but from a recognition of women's potential within our academic collective. Only when women reach senior positions empowered rather than already depleted—not having had to paddle against the tide while bailing water—will we have the ability to (finally) behead the sexist monster that guards the doors to academia.

## Author Biography

Olivia del Giorgio (olivia.delgiorgio@mail.mcgill.ca) is affiliated with the Department of Geography at McGill University, in Montreal, Quebec, Canada. Gabriela Fontanarrosa is affiliated with the Institute of Neotropical Biodiversity at Tucumán National University and CONICET, in Tucumán, Argentina. Silvia Lomáscolo is affiliated with the Institute of Regional Ecology at Tucumán National University and CONICET, in Tucumán, Argentina. María Piquer-Rodríguez is affiliated with the Institute of Geographical Sciences at Freie Universität, in Berlin, Germany.
